# An in vitro biomechanical evaluation of an expansive double-threaded bi-directional compression screw for fixation of type II odontoid process fractures: A SQUIRE-compliant article

**DOI:** 10.1097/MD.0000000000006720

**Published:** 2017-04-21

**Authors:** Ning Liu, Li Tian, Rong-Xian Jiang, Chao Xu, Lei Shi, Wei Lei, Yang Zhang

**Affiliations:** a Department of Orthopedics, Xijing Hospital, The Fourth Military Medical University; b Department of Anesthesiology, Xijing Hospital, The Fourth Military Medical University, Xi’an; c Department of Orthopedics, 62th Hospital of PLA, Puer, China.

**Keywords:** biomechanics, bone screws, internal fixator, odontoid process fracture, shear stiffness, tensile stiffness, torsional stiffness

## Abstract

Odontoid process fracture accounts for 5% to 15% of all cervical spine injuries, and the rate is higher among elderly people. The anterior cannulated screw fixation has been widely used in odontoid process fracture, but the fixation strength may still be limited under some circumstances. This study aims to investigate the biomechanical fixation strength of expansive double-threaded bi-directional compression screw (EDBCS) compared with cannulated lag screw (CLS) and improved Herbert screw (IHS) for fixation of type II odontoid process fracture.

Thirty fresh cadaveric C2 vertebrae specimens were harvested and randomly divided into groups A, B, and C. A type II fracture model was simulated by osteotomy. Then the specimens of the 3 groups were stabilized with a single CLS, IHS, or EDBCS, respectively. Each specimen was tested in torsion from 0° to 1.25° for 75 s in each of 5 cycles clockwise and 5 cycles anticlockwise. Shear and tensile forces were applied at the anterior-to-posterior and proximal-to-distal directions, respectively, both to a maximum load of 45 N and at a speed of 1 mm/min.

The mean torsional stiffness was 0.309 N m/deg for IHS and 0.389 N m/deg for EDBCS, which were significantly greater compared with CLS, respectively (0.169 N m/deg) (*P* < .05 and *P* < .05). The mean shear stiffness for the EDBCS was 238 N/mm, which was significantly greater than CLS (150 N/mm) and IHS (132 N/mm) (*P* < .05 and *P* < .05). All 3 screws only partly restored tensile stiffness, but not significantly.

Fixation with the EDBCS can improve the biomechanical strength for odontoid process fracture compared with CLS and IHS, especially in terms of torsional and shear stiffness.

## 1 Introduction

Odontoid process fracture accounts for 5% to 15% of all cervical spine injuries, and the rate is higher among elderly people.^[1,2]^ Type II odontoid process fracture is the most common odontoid injury, which can cause atlantoaxial instability.^[3]^ Moreover, type II odontoid process fracture can hardly healing spontaneously, and its union rate is lower compared with types I and III fractures.^[4–6]^ Nonoperative treatment with a rigid brace can facilitate fracture healing, but the mortality rate among elderly patients is 26% to 47%.^[7]^ Such high mortality rates are probably caused by respiratory-related complications due to long-term external immobilization.^[8]^ The union rates were 92.8% to 100% among patients undergoing traditional posterior C1–C2 arthrodesis.^[3,9]^ A major untoward consequence of this technique is the decreased cervical motion: movement at C1–C2 accounts for more than 50% of all cervical spine rotatory motion and about 10% of all cervical spine flexion–extension.^[10]^

Direct anterior screw fixation has been used to stabilize type II odontoid process fracture since early 1980s.^[11]^ This technique provides immediate stabilization with minimal external support postoperatively,^[4,6,12–15]^ and allows for good anatomical and functional restoration, with high union rates (90–100%).^[7,14,16–22]^ Many types of screws have been used to treat type II odontoid process fracture, including Herbert screws,^[4,23,24]^ Knoeringer double-threaded screws,^[25]^ cortical^[17,20]^ or cancellous bone screws,^[16,23]^ fully,^[17,20]^ partially^[19,20,22]^ threaded or lag screws,^[7,20,21,23]^ cannulated screws,^[14–16]^ and self-tapping^[13,17,18]^ or nonself-tapping screws.^[15,20,21]^ They are made of stainless steel or titanium^[15,17,21]^ with diameter of 2.7,^[17]^ 3.0,^[22]^ 3.5,^[13,18,20,23]^ 4.0,^[15,16]^ or 4.5 mm.^[16,19,22–24]^ These screws may have different biomechanical properties, but fixation with a single cannulated lag screw (CLS) is accepted generally.^[7,16,26–28]^ However, the usage of these screws is still limited by the weak fixation strength under some circumstances. The odontoid process also may rotate if the fixation screw is loose or cuts out of the C2 vertebrae body.

Bone mineral density (BMD) as 1 major quantitative parameter of bone quality can affect the screw's stability.^[29–31]^ In addition, BMD is not uniformly distributed in the axis: the density is higher at the tip of the odontoid but lower in the neck of the axis, and the cortical bone density is high in the anteroinferior region of C2.^[32]^ Screw loosening, cut-out, displacement, and other incidents commonly occur in elderly patients because of the presence of osteoporosis and the weak holding ability of screws to the bone.^[33]^

To solve these problems, we designed an expansive double-threaded bi-directional compression screw (EDBCS) based on the IHS.^[22,24,28,34]^ The EDBCS combines the features of expansive pedicle screw,^[35]^ expansive cannulated screw,^[36]^ and CLS. The principal purpose of this study is to compare the mechanical torsional, shear and tensile stiffness of the EDBCS with those of the conventional screws including CLS and IHS, and to evaluate the mechanical properties for fixation of type II odontoid process fractures.

## 2 Materials and methods

### 2.1 Implant description

**Figure 1:**
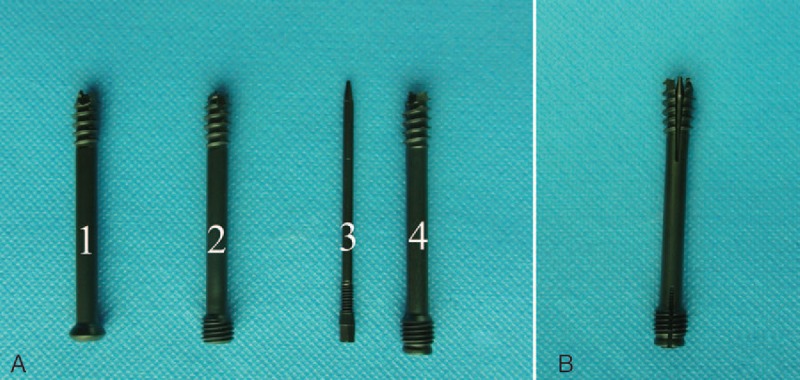
Three kinds of screws. (A) All the 3 kinds of screws. (1) Cannulated lag screw. (2) Improved Herbert screw. (3) The internal smaller gauge screw of the expansive double-threaded bi-directional compression screw (EDBCS). (4) The external part of EDBCS. (B) The expanding EDBCS.

The double-threaded, cannulated, self-tapping, and self-drilling Herbert screw was made of Ti6Al4V. We improved its thread design by introducing varying diameters and pitches at different sections. The thread pitches at the proximal end are twice finer than at the distal end (Fig. 1A). This thread profile can produce compression between fracture fragments when the screw is driven. The diameters of the distal and the proximal thread portions are 4.0 and 4.5 mm, respectively. Both threads during insertion can engage with the bone with maximal fixation strength.

The EDBCS consists of 2 titanium alloy components: a cannulated screw and a gauge screw. The gauge screw can be inserted into the cannulated screw to open the fins on both ends (Fig. 1A). The pitches at the proximal end are finer than IHS at the distal end. The diameter augments at the screw tip and end are 0.9 and 0.8 mm, respectively. The diameter of the screw's middle portion remained constant (Fig. 1B).

### 2.2 Ethics statement and specimen preparation

This study and the consent procedure were approved by the Institutional Review Broad of Fourth Military Medical University, Xi’an, Shaanxi Province, China. We did not conduct our research outside our country of residence and obtained written informed consent from all participants’ relatives involved in our study.

Thirty fresh human cadaveric C2 specimens aged 48 to 78 (mean 67) years old were used in this study. They were all males and of Chinese origin. After dissection of all soft tissues and cartilage, anteroposterior and lateral view radiographs were obtained to rule out the possibility of abnormalities or fractures. Then the specimens were double-bagged, stored in a freezer at −20°C until 24 h before testing, and then thawed at room temperature.^[26,37]^

### 2.3 Bone mineral density

BMD was scanned at 3 levels for each specimen: on the top, base of the odontoid, and on the anteroinferior part of the axis using a dual energy X-ray absorption meter (Lunar Corp., Madison, WI). The mean BMD of the 3 levels was regarded as the BMD of the axis.^[26]^

### 2.4 Biomechanical tests

**Figure 2:**
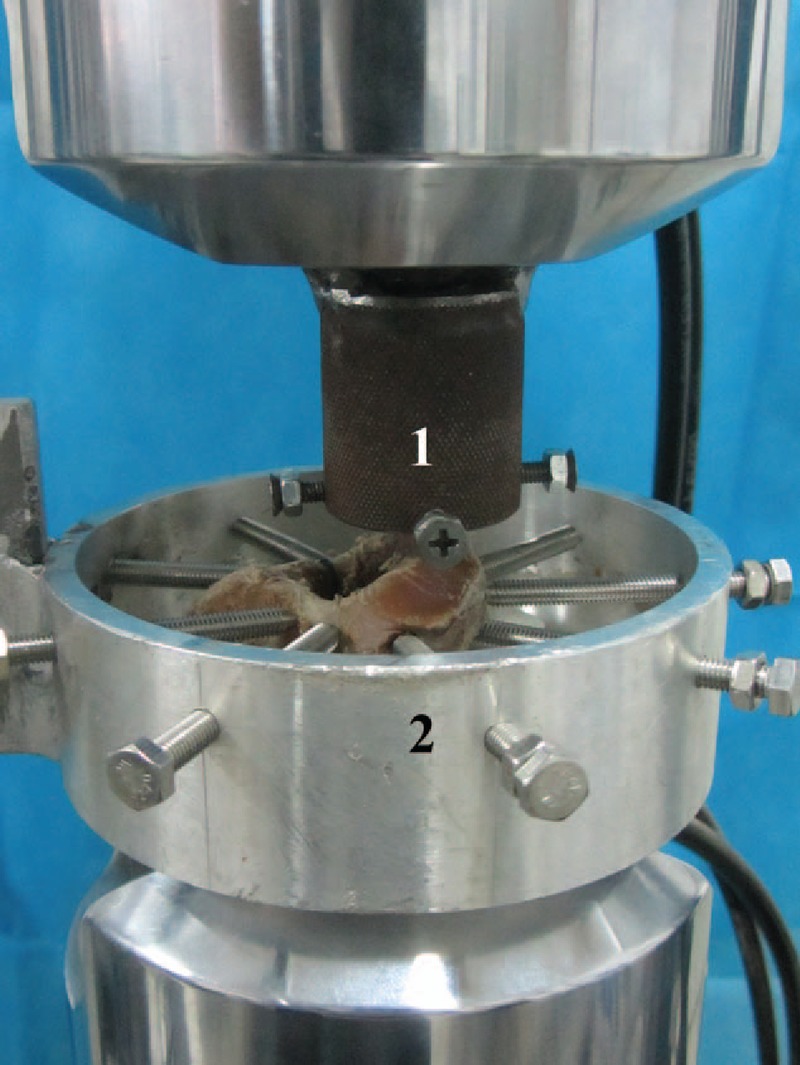
Testing of torsional stiffness and tensile stiffness. (1) The ferruginous ring equipped with 4 threaded positioning rods. Each rod was partially sharpened at the tip to securely fix the odontoid process. (2) An aluminum alloy ring with 9 threaded positioning rods designed to securely fix the body of the C2 vertebrae.

The C2 specimens were divided randomly into 3 groups (A, B, and C, each n = 10). The mean ages of the donors and the BMD of the specimens were not different between the 3 groups. The specimens of groups A, B, and C were fixed with CLS, IHS, and EDBCS, respectively. The odontoid process was placed inside a metal ring with 4 threaded positioning rods and thereby rigidly affixed to the testing machine. Each rod had a sharp tip for securely fixation of the odontoid process. The C2 vertebrae were fixed using a similar but larger metal ring (Fig. 2).^[28,37]^

Torsional test was performed on material testing system (MTS) 858 (MTS System Inc., Minneapolis, MN). Each specimen was rotated from 0° to 1.25° in 75 s in each of 5 cycles clockwise and 5 cycles anticlockwise. There was a break of 1 min between every 2 cycles.^[26,28,38–41]^ The torsional stiffness was evaluated from the slope of the “torque versus rotation angle” curve for each cycle, and then the values of all the cycles were averaged.

**Figure 3:**
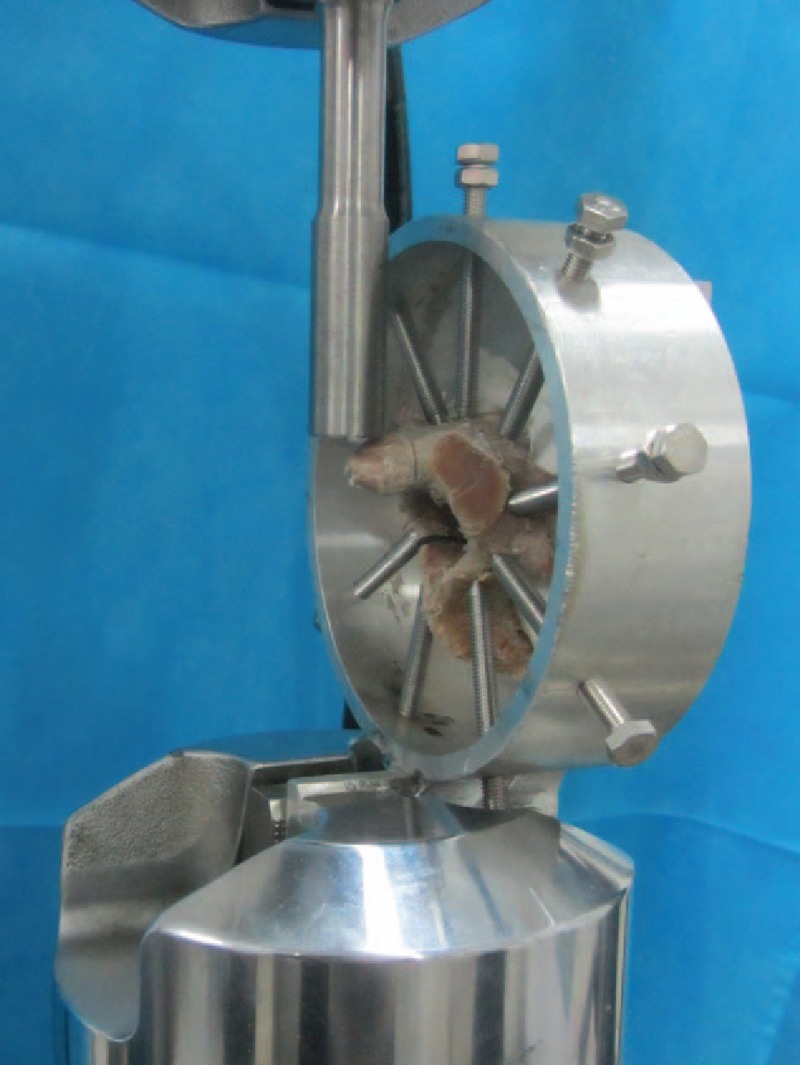
Testing of shear stiffness.

Shear force was applied to the odontoid from the anterior-to-posterior direction on MTS 858. The odontoid was loaded at a displacement rate of 1 mm/min to a maximum load of 45 N in each of 5 cycles. There was a break of 1 min between every 2 cycles.^[26,28,42,43]^ The shear stiffness was calculated from the slope of the “force versus displacement” curve, and then the values of all cycles were averaged (Fig. 3).

Tensile force was applied to the odontoid axially from the proximal-to-distal direction on MTS 858. The specimens were mounted in the same position as in the torsional test. The odontoid was loaded at a displacement rate of 1 mm/min to a maximum load of 45 N in each of 5 cycles. There was a break of 1 min between every 2 cycles.^[9,10,41,44–46]^ The tensile stiffness was calculated from the slope of “force versus displacement” curve, and then the values of all cycles were averaged. The torque, force, and displacement data were recorded throughout the study at a frequency of 10 Hz.

### 2.5 Osteotomy

In each specimen, 1 guide wire was placed parallel to the coronal plane from the anterior–inferior lip of the vertebrae body into the posterior–superior portion of the odontoid. After the hole was drilled and tapped, an osteotomy was performed at the junction of the odontoid process and C2 body and a thin oscillation saw was used to simulate a type II fracture pattern^[28,44]^ according to the classification of Anderson and D’ Alonzo.^[47]^

### 2.6 Screw fixation

**Figure 4:**
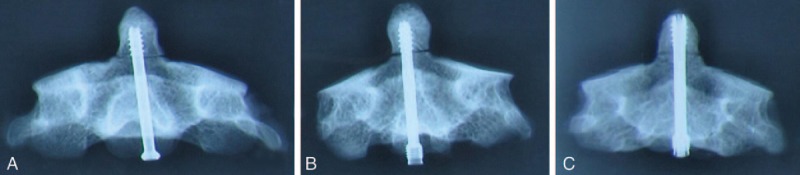
X-ray after screw fixation. (A) Fixed with cannulated lag screw. (B) Fixed with improved Herbert screw. (C) Fixed with expansive double-threaded bi-directional compression screw.

One experienced surgeon performed the screws fixation. The resected odontoid was instrumented with a CLS, IHS, or EDBCS of appropriate length. After the EDBCS cannulated screw was inserted, both of its proximal and distal ends were expanded by threading the smaller gauge screw into the inner core. All screws were inserted without perforating the odontoid's apical cortex. Anterior–posterior and lateral radiographs were obtained for each specimen to verify the screw placement and to measure the diameters at the tips and ends of EDBCS (Fig. 4). The specimens were mounted for test of stiffness at the 3 directions.

### 2.7 Statistical analysis

Statistical comparisons were performed on SPSS 19.0 (SPSS Inc., Chicago, IL). The results were expressed as mean ± standard deviation. The effects of BMD on stiffness were assessed using Pearson correlation. The results of stiffness among groups before fixation were compared using analysis of variance (ANOVA). The effects of fixation with screws on torsional stiffness, shear stiffness, and tensile stiffness within a group were evaluated using paired *t* test. The fixation strengths among groups were compared using 2-sample *t* test.

## 3 Results

The diameters of the expandable parts of EDBCS in group C were measured by radiographs. The diameter ranged from 4.54 to 4.86 (mean 4.75) mm at the distal end and from 5.20 to 5.38 (mean 5.24) mm at the proximal end. The diameters of the distal and proximal ends of the EDBCS before expansion were 4.0 and 4.5 mm, respectively.

**Figure 5:**
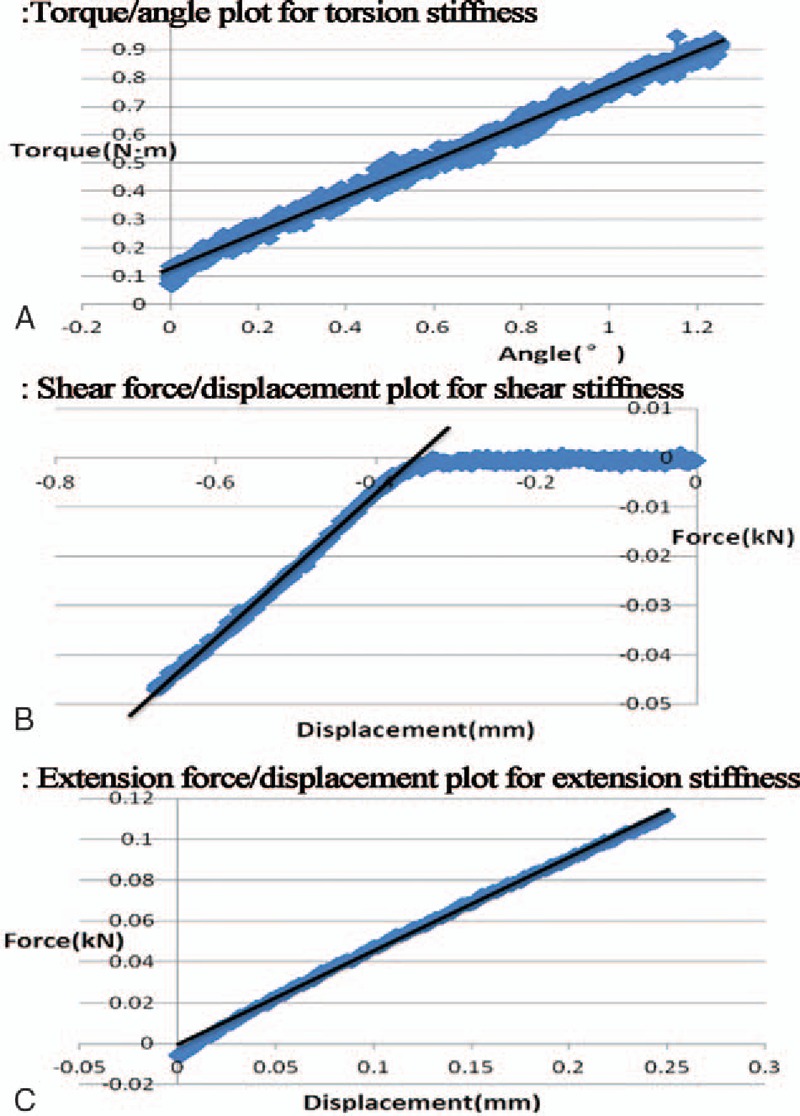
Plots for stiffness. Note the slope of black lines (trend lines) originating from initial torque/angle (A) or force/displacement (B, C) curve representing correspondent stiffness. Blue points represent the data collected by the computer data acquisition system attached to the testing machine.

The mean stiffness of the intact and instrumented specimens of each group was calculated from the slopes of the “torque/force versus angle/displacement” curves (Fig. 5). Torsional and shear stiffness of the instrumented odontoid significantly decreased in both groups A and B. Shear stiffness was restored in group C. The restoration ratio of mean torsional stiffness of instrumented odontoid was <55% in all 3 groups. The mean tensile stiffness of the instrumented specimens increased by about 10%, but not significantly (Table 1).

**Table 1:**
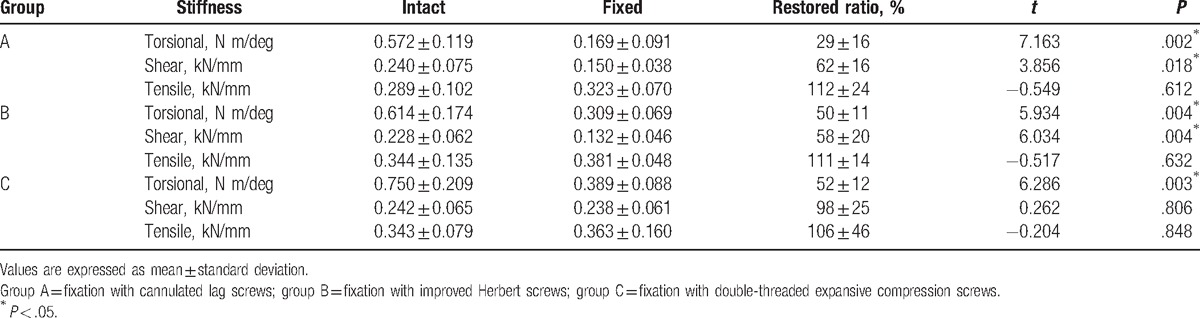
Mean stiffness of specimens before and after fixation and their difference between them.

The BMDs of the specimens ranged from 0.533 to 0.792 (0.667 ± 0.084) g/cm^2^ and were not significantly different among groups (ANOVA, *P* = .164) (Table 2). BMD did not correlate with any type of stiffness before or after screw fixation in each group (*P* > .05) (Table 3). All 3 types of stiffness before screw fixation were not different among groups (Table 4). The results of torsional stiffness after fixation were significantly different between groups A and B, and between groups A and C (*P* < .05). The results of shear stiffness were significantly different between groups A and C, and between groups B and C (*P* < .05). The results of tensile stiffness were not significantly different between groups (Table 5).

**Table 2:**
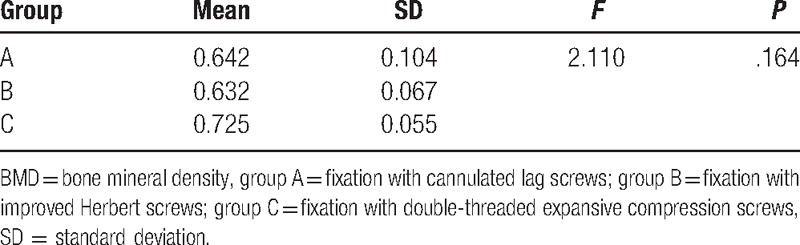
BMD (g/cm^2^) of the specimens.

**Table 3:**
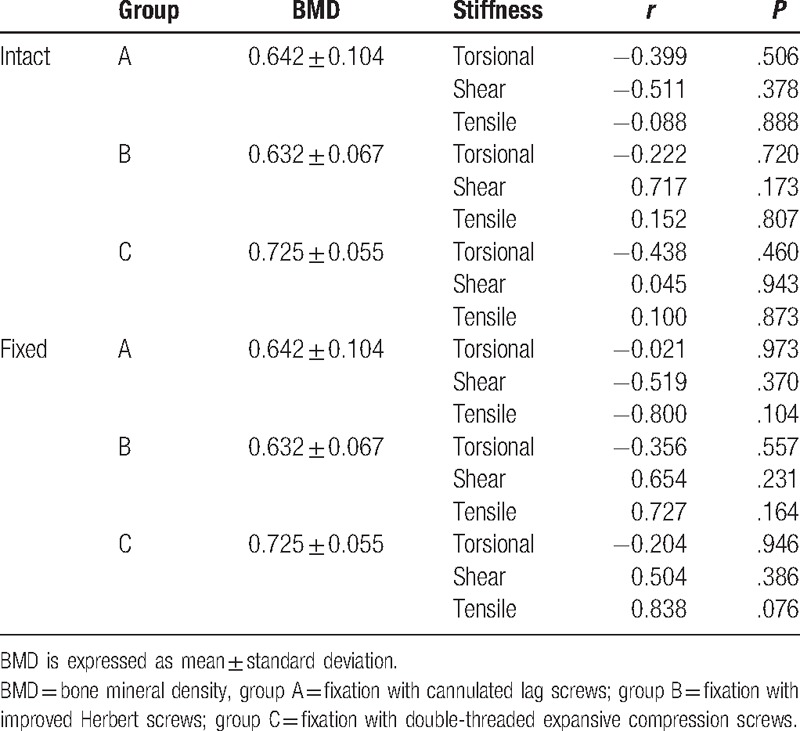
Correlation analysis between BMD (g/cm^2^) and stiffness before and after screw fixation.

**Table 4:**
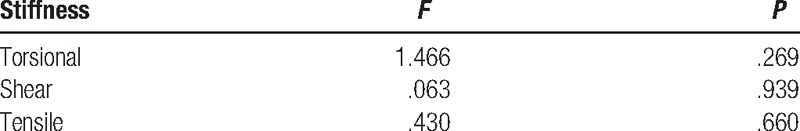
Analysis of variance of mean stiffness before screw fixation.

**Table 5:**
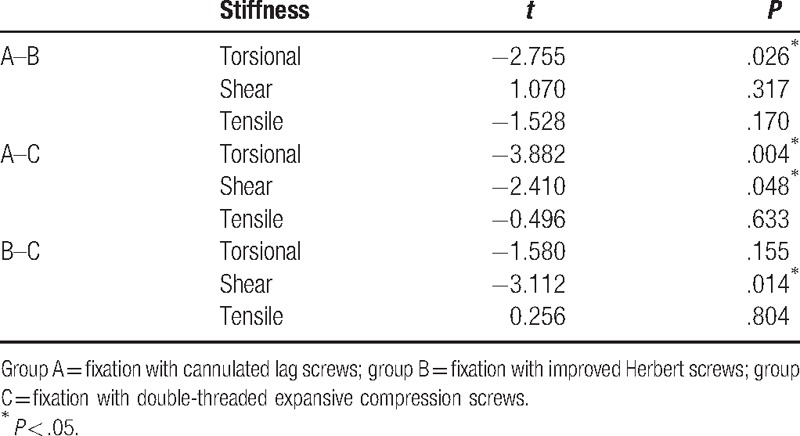
Two-sample *t* test of mean stiffness after fixation between groups.

## 4 Discussion

The use of anterior CLS in odontoid process fracture fixation has been well accepted. Morphologic studies suggest that the diametric dimensions of the odontoid in most patients are insufficient to accommodate only 1 but not 2 screws with diameter ≥3.5 mm.^[19,38]^ As the primary control group, we used CLS with diameter of 4.0 mm, which is also used in our hospital. We also chose IHS as another control group because its fixation strength was well proven.^[22,24,28]^ Herbert screw was also widely used for fixation of scaphoid bone fracture,^[48,49]^ it was improved and had been applied to odontoid process fracture with high stability.^[22,24,28,34]^ However, the fixation strength was weak^[34,50]^ and the stiffness restoration ratio was <50% intact specimens.^[28]^

Despite the excellent results with CLS, pseudarthrosis rates were also up to 20%.^[27,30]^ Patients with pseudarthrosis experienced loss of fixation more frequently in the C2 body than in the odontoid.^[7,16]^ Biomechanical studies confirmed these results.^[51,52]^ This failure mode is consistent with the osseous structure of the C2 body, which has lower cortical thickness and lower bone density.^[53–55]^ Thus, research focused on improving bony purchase within the C2 body.^[7,16,51,52,54–56]^ A hybrid locking plate/variable pitch screw construct for anterior fixation of type II odontoid process fracture was used to strengthen the purchase within the C2 body. But this construct was much more complicated.^[44]^ In our study, IHS and EDBCS can improve the effective contact between the screw threads and the vertebra's cortical bone compared with CLS. We may infer that IHS and EDBCS can prevent the screw's proximal end from cutting out through the anteroinferior part of C2 body and decrease the pseudarthrosis rates.

BMD could influence the screw's holding ability in the bone.^[29–31]^ To account for possible variation in bone quality, we divided the specimens randomly into 3 groups with no difference in BMD. However, we found that BMD was not correlated with the odontoid stiffness either before or after screw fixation (*P* > .05).

Fixation with IHS and EDBCS outperformed CLS in terms of torsional stiffness. The larger torsional stiffness may be attributed to the larger compressive force between the fracture fragments (which improved the frictional force between fragments so as to resist torsional movement),^[28]^ and also the interaction between the screw threads and the axis’ cortical bone. The expandable ends of EDBCS may further contribute to higher torsional stiffness. In fact, the torsional stiffness of EDBCS was larger than IHS though not significantly. The shear stiffness was not significantly different between CLS and IHS, but the shear stiffness of EDBCS was greater than both CLS and IHS. Hence, our study demonstrates that the EDBCS provides higher fixation strength in terms of transverse shear compared with the widely used CLS and IHS.

The compression generated between the 2 fracture fragments after insertion of screws contributes to bone healing. The compression can be evaluated with tensile stiffness or load to failure.^[43]^ Both measurement methods can be used to test the stability of type II odontoid facture. In our study, only tensile stiffness was measured. Load to failure was not used because the stiffness-provided initial stability was the major parameter before fracture healing.^[28]^ Furthermore, a nondestructive low load test allowed us to complete all mechanical tests on each specimen. The selected load was about half of the physiologic load and was enough to obtain the torque-angle and load-linear displacement curves.^[26,28]^ All 3 types of fixation methods, CLS, IHS, and EDBCS, provided slightly greater tensile stiffness than the intact specimens, although the results were not significant (*P* > .05). Even the EDBCS did not provide greater compression force to the fracture fragments.

One limitation of the present study was that to establish a reproducible fracture model, an osteotomy with a thin oscillation saw at the junction of the odontoid process and the vertebra was performed to simulate a type II fracture pattern. However, such a smooth horizontal fracture line may not occur in patients.

## 5 Conclusions

In summary, we concluded that the use of an EDBCS can improve the biomechanical fixation strength for type II odontoid process fracture compared with CLS and IHS, especially in terms of torsional and shear stiffness. However, the experimental data may not be applicable to clinical outcomes and thus should be confirmed via prospective clinical studies in vivo.

## Acknowledgments

The authors thank the State Key Laboratory for Strength and Vibration of Mechanical Structures, School of Mechanical Engineering, Xi’an Jiao Tong University for research and institutional support.
